# Supplementary material for: Evaluation of NV556, a Novel Cyclophilin Inhibitor, as a Potential Antifibrotic Compound for Liver Fibrosis
Source: Cells. 2019 Nov 8;8(11):1409. doi: 10.3390/cells8111409 (PMC6912624; doi:10.3390/cells8111409)
Supplement: Supplementary file 1 [file cells-08-01409-s001.pdf]

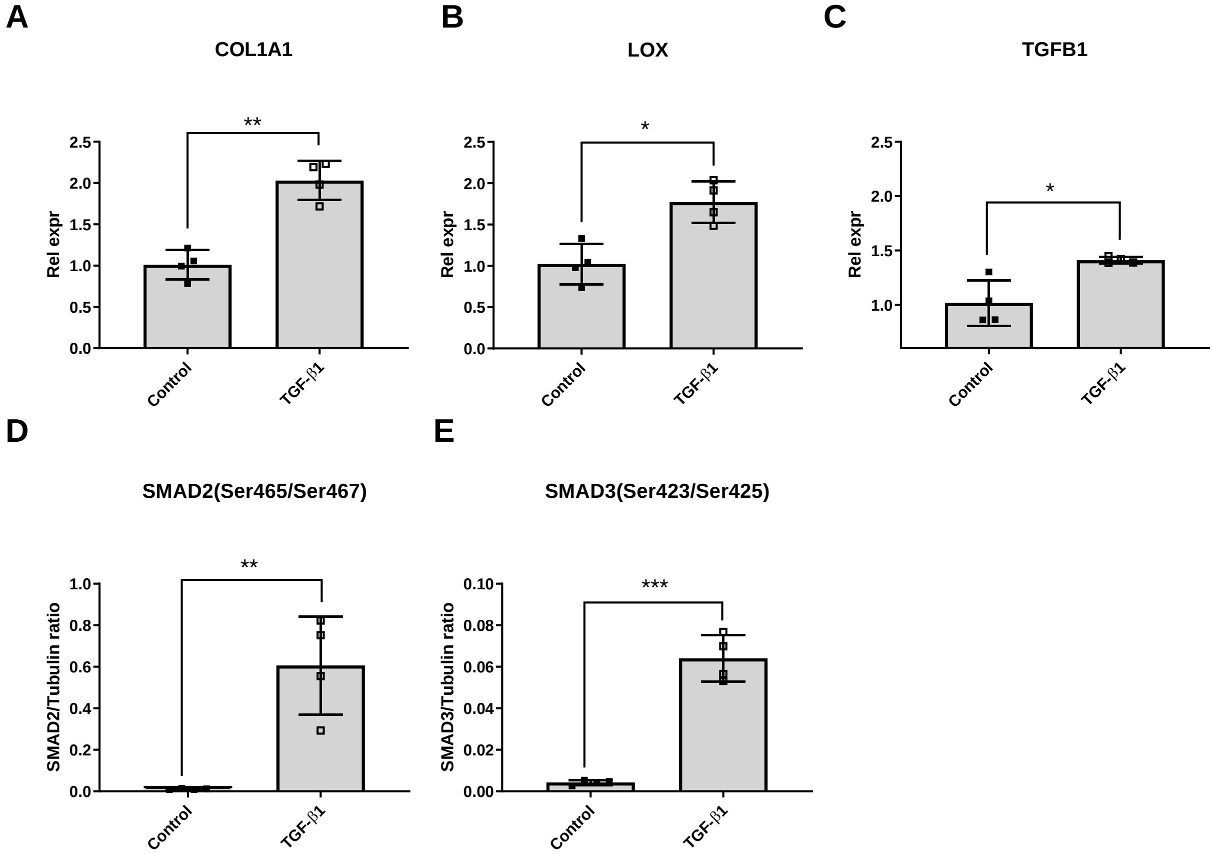

Figure S1. TGF-β1 effect on gene expression in comparison to control (A-C), and of SMAD2 (D) and SMAD3 (E) phosphorylation levels, in a 3D human liver model reseeded with LX2 cells. Data in A-C are represented as mean of relative expression over inactive control ± SD and Data in A-E are statistically analyzed by a paired T-test. *$p < 0.05$, **$p < 0.01$, ***$p < 0.001$. n=4 scaffolds per condition investigated

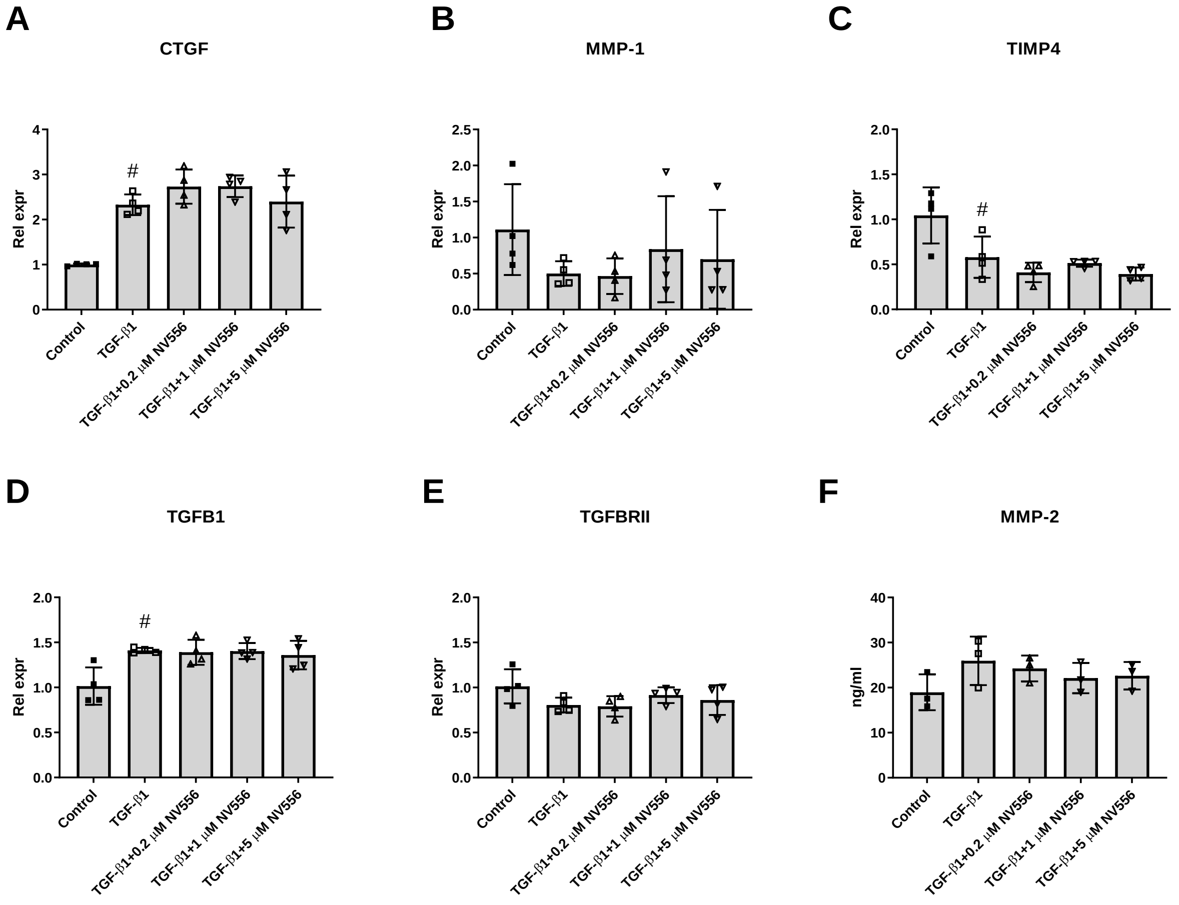

Figure S2. NV556 effect on gene expression (A-E) and protein secretion (F) in a 3D human liver model reseeded with LX2 cells. Data in A-E are represented as mean of relative expression ± SD and Data in A-F are statistically analyzed by one-way ANOVA followed by Tukey's multiple comparison test for activated LX2. #p <0.05 for TGF-β1 versus non-treated Control cells. n=4 scaffolds per condition investigated

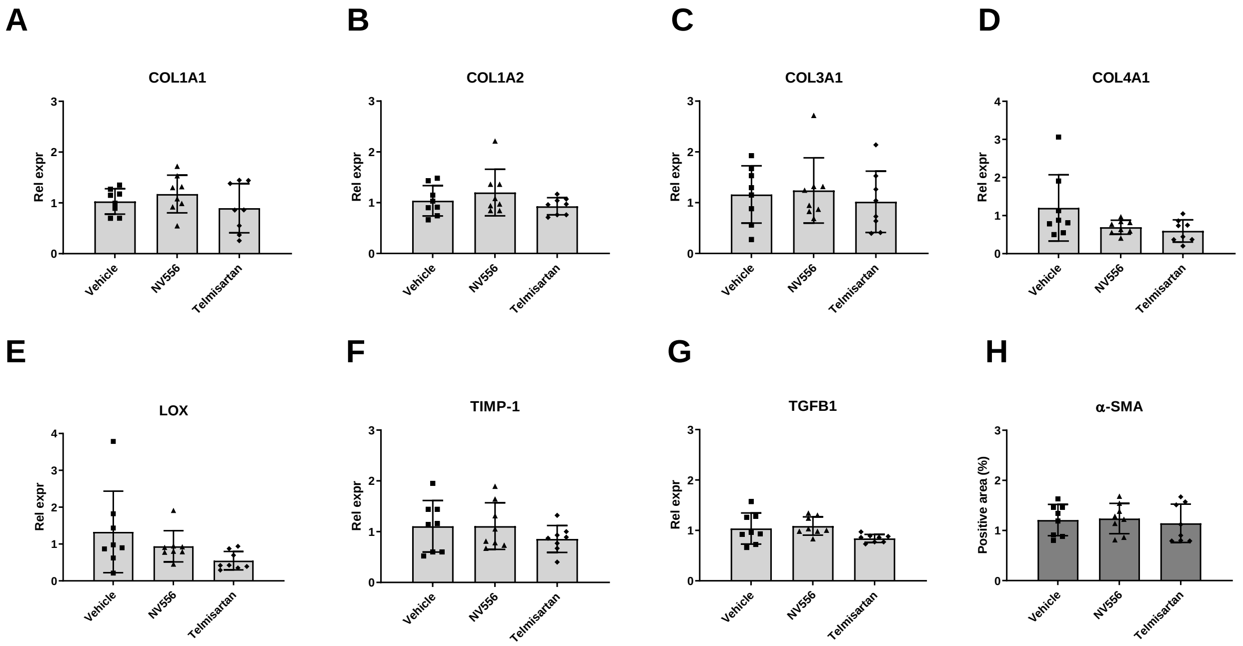

Figure S3. NV556 effect on gene expression (A-G) and αSMA positive area (H) in the STAM model. Data in A-G are represented as mean of relative expression ± SD and Data in A-H are statistically analyzed by one-way ANOVA followed by Dunnett's multiple comparison test. n=8 mice per group
